
JOURNAL INFORMATION
==============================
NLM Title Abbreviation: ScientificWorldJournal
Journal ID: ScientificWorldJournal
NLM Journal ID: 101131163
Journal ID: TSWJ

The Scientific World Journal

ISSN: 2356-6140
EISSN: 1537-744X
Publisher: Wiley

ARTICLE INFORMATION
==============================
PMCID: PMC5956415
PMID: 15349532
DOI: 10.1100/tsw.2004.51
Article ID: 784585
Article version: 1
Subjects: Case Report

Adenomatous Polyp of the Verumontanum Causing Bladder Outlet Obstruction

Daneshmand Siamak
Department of Urology, USC/Norris Comprehensive Cancer Center, Los Angeles, CA, USA
Publication date: 2004
Electronic publication date: 2004 Jun 7
Volume: 4
First page: 89
Last page: 91
Copyright: Copyright © 2004 Siamak Daneshmand.
Copyright year: 2004
License: This is an open access article distributed under the Creative Commons Attribution License, which permits unrestricted use, distribution, and reproduction in any medium, provided the original work is properly cited.
License URL: https://creativecommons.org/licenses/by/3.0/

==============================
Case Report
*TheScientificWorldJOURNAL* (2004) 4 (S1), 89–91
ISSN 1537-744X; DOI 10.1100/tsw.2004.51

### Adenomatous Polyp of the Verumontanum Causing Bladder Outlet Obstruction

Siamak Daneshmand, MD
*Department of Urology, USC/Norris Comprehensive Cancer Center, Los Angeles, CA*

**E-mail**: siadaneshmand@yahoo.com

*Previously published in the Digital Urology Journal*

**DOMAIN**: urology

### CASE REPORT

A 27-year-old male presented with a history of gross hematuria with passage of clots. In addition to suprapubic pain with urination, he reported intermittency, urgency, frequency, and a sense of incomplete emptying. Valsalva or physical compression of his lower abdomen was necessary to pass urine. An outpatient intravenous pyelogram was negative, but retrograde urethrography demonstrated a filling defect at the base of the bladder (Figure 1). Flexible cystourethroscopy revealed a 3 cm pedunculated smooth polypoid mass arising from the verumontanum and projecting proximally into the bladder neck, causing a ball-valve effect. The patient underwent a transurethral resection of the mass. Histopathologic examination revealed a polypoid mass lined by a mixture of transitional and squamous epithelium composed of benign prostatic glands and stroma (Figure 2). The prostatic glands showed varying degrees of dilatation and hyperplasia and contained numerous corpora amylacea. Immunoperoxidase staining showed the epithelial cells of the glands to be strongly positive for prostate-specific antigen and prostate-specific acid phosphatase (Figure 3). Postoperatively the patient reported complete resolution of obstructive urinary symptoms as well as an increase in his ejaculatory volume. He has since experienced no recurrence of his symptoms.

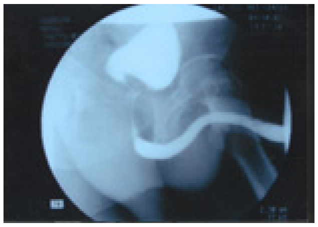

**FIGURE 1**. Retrograde urethrogram identifying mass at the bladder neck.

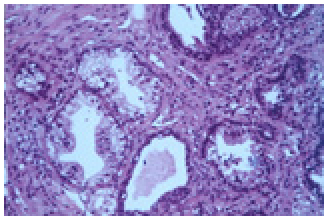

**FIGURE 2**. Histopathology of mass. Note benign prostatic glands and stroma.

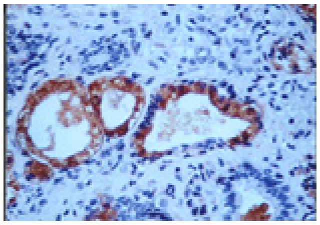

**FIGURE 3.** Immunoperoxidase staining showing strong positivity for prostate specific antigen and prostate-specific acid phosphatase.

### DISCUSSION

Benign prostatic urethral polyps are uncommon, and rarely present in adulthood. They most frequently arise in the prostatic fossa, and are generally thought to represent a developmental anomaly in the invagination of glandular tissue of the inner zone of the prostate.(1) These polyps frequently present with symptoms of bladder outlet obstruction, including frequency and urgency, and with hematuria or hematospermia. Occasionally, acute urinary retention is the presenting complaint. Clinical examination, urinalysis, and urine culture are typically normal, and intravenous urography is often negative. In addition to the symptoms listed above, complications of bladder outlet obstruction may develop including urinary tract infection, hydronephrosis, hydroureter, and urolithiasis.(2) Ultrasonography, excretory urography, and voiding cystourethrography with fluoroscopy have been found to be effective in identifying urethral polyps, while cystourethroscopy serves to both confirm diagnosis and provide therapy via transurethral resection of the mass, which is the treatment of choice.(3) Other options for treatment include polyp excision by open cystotomy or endoscopic excision using laser energy. After excision by fulguration, resection, or laser excision, symptoms usually resolve. Patients who may have been experiencing a component of retrograde ejaculation secondary to the polyp may experience an increase in apparent volume of ejaculation. Recurrence of the polyp after complete resection is extremely rare.(4) The diagnosis of urethral polyp should be considered in young male patients presenting with gross hematuria, hematospermia, or symptoms of bladder outlet obstruction in the face of an otherwise benign clinical picture.

### REFERENCES

1. Walsh IK, Keane PF, Herron B: Benign urethral polyps. Br J Urol, 1993, 72(6):937.
2. Sekido N, Hinotsu S, Akaza H, Koiso K: Fibroepithelial polyp of the prostatic urethra: report of two cases and review of the literature. Jap J Urol, 1994, 85(9):1403,.
3. Spyropoulos C, Konidaris D, Papanicolaou A, Stephanidis A, Michael V, Androulakakis PA: Posterior urethral polyp in a boy, diagnosed by colour Doppler ultrasonography. BJU Int, 1999, 84:881.
4. Anjum MI, Ahmed M, Shrotri N, Azzopardi A, Mufti GR: Benign polyps with prostatic-type epithelium of the urethra and the urinary bladder. Int Urol Nephrol, 1997, 29(3):313.

**This article should be referenced as follows:**

Daneshmand, S. (2004) Adenomatous polyp of the verumontanum causing bladder outlet obstruction. *TheScientificWorldJOURNAL* **4 (S1)**, 89–91.

**Handling Editor:**

Anthony Atala, Principle Editor for *Urology* — a domain of *TheScientificWorldJOURNAL*.
